# Daratumumab monotherapy in a patient with kappa light chain multiple myeloma and decompensated cirrhosis: a case report

**DOI:** 10.3389/fonc.2025.1696746

**Published:** 2025-12-12

**Authors:** Xiying Yang, Zhiqian Liu, Yangyan Zhou, Hua Zhang, Min Yang, Hong Wang

**Affiliations:** 1 Department of Gastroenterology, Digestive Disease Hospital, Affiliated Hospital of Zunyi Medical University, Zunyi, Guizhou, China; 2 Department of Hematology, Affiliated Hospital of Zunyi Medical University, Zunyi, Guizhou, China

**Keywords:** multiple myeloma, liver cirrhosis, light chain deposition, serum free light chain, daratumumab, case report

## Abstract

**Background:**

Multiple myeloma (MM) rarely presents as decompensated liver cirrhosis, posing a significant diagnostic challenge that can lead to misdiagnosis and delayed treatment. We report a case of kappa-light chain multiple myeloma (κ-LCMM) initially managed as end-stage liver disease to highlight the diagnostic pathway and a successful, organ-reversing therapeutic outcome.

**Case presentation:**

We report the case of a 67-year-old male who presented with decompensated liver cirrhosis and refractory ascites as the initial manifestation of multiple myeloma. Serum free light chain assay revealed a kappa level of 47,309.88 mg/L, and bone marrow biopsy confirmed kappa-light chain multiple myeloma with 60% plasma cell infiltration. A profound clinical response, including reversal of hepato-renal dysfunction, was observed following targeted monotherapy with daratumumab.

**Conclusions:**

This case underscores the critical importance of including plasma cell dyscrasias in the differential diagnosis of unexplained cirrhosis, particularly when accompanied by decreased immunoglobulin levels. The serum free light chain assay is an essential tool for facilitating a timely diagnosis. Furthermore, our findings suggest that daratumumab monotherapy can be a safe and highly effective therapeutic strategy for achieving deep, durable responses and reversing severe organ damage in patients with multiple myeloma.

## Introduction

This report delineates the diagnostic and therapeutic course of a patient with liver cirrhosis secondary to kappa-light chain multiple myeloma (κ-LCMM), whose initial presentation was dominated by gastrointestinal manifestations. Upon admission, the patient was found to have cirrhosis of unknown etiology. After a comprehensive workup excluded common causes such as viral hepatitis, alcoholic liver disease, and autoimmune hepatitis, serum free light chain analysis revealed a marked elevation in monoclonal kappa chains. The definitive diagnosis of κ-LCMM was established by bone marrow pathology. Following daratumumab therapy, we observed in a significant amelioration of hepato-renal function, complete resolution of refractory ascites, and a quantifiable reduction in liver stiffness as measured by transient elastography. These findings strongly suggest that the patient’s cirrhosis was a direct consequence of the hepatic deposition of monoclonal immunoglobulin light chains secreted by the neoplastic plasma cell clone. Consequently, this case underscores the imperative for clinicians to include plasma cell dyscrasias within the differential diagnosis of cirrhosis of indeterminate etiology. Prompt screening with serum free light chain assays and bone marrow examination is paramount for facilitating early diagnosis, optimizing therapeutic response, and ultimately, improving patient prognosis.

## Case presentation

A 67-year-old male was admitted to our institution on June 18, 2024, presenting with a one-month history of nausea and vomiting, and progressive abdominal distension over the preceding 10 days. One month prior, the patient had sought medical attention at an outside hospital for similar symptoms, where laboratory investigations revealed elevated serum transaminases. He was subsequently discharged following symptomatic improvement with hepatoprotective therapy. Ten days prior to the current admission, these symptoms recurred and were exacerbated, now accompanied by abdominal pain. At a local hospital, a diagnostic workup excluded Hepatitis B and C, while tumor markers, including alpha-fetoprotein (AFP) and carcinoembryonic antigen (CEA), were within normal limits. An abdominal ultrasound demonstrated a massive volume of ascitic fluid. Despite therapeutic paracentesis and ongoing hepatoprotective treatment, the patient’s condition showed no amelioration, and he was consequently transferred to our institution for further investigation and management. The patient denied any history of chronic alcohol consumption or exposure to known hepatotoxic agents. His past medical and family histories were otherwise non-contributory.

**Figure 1:**
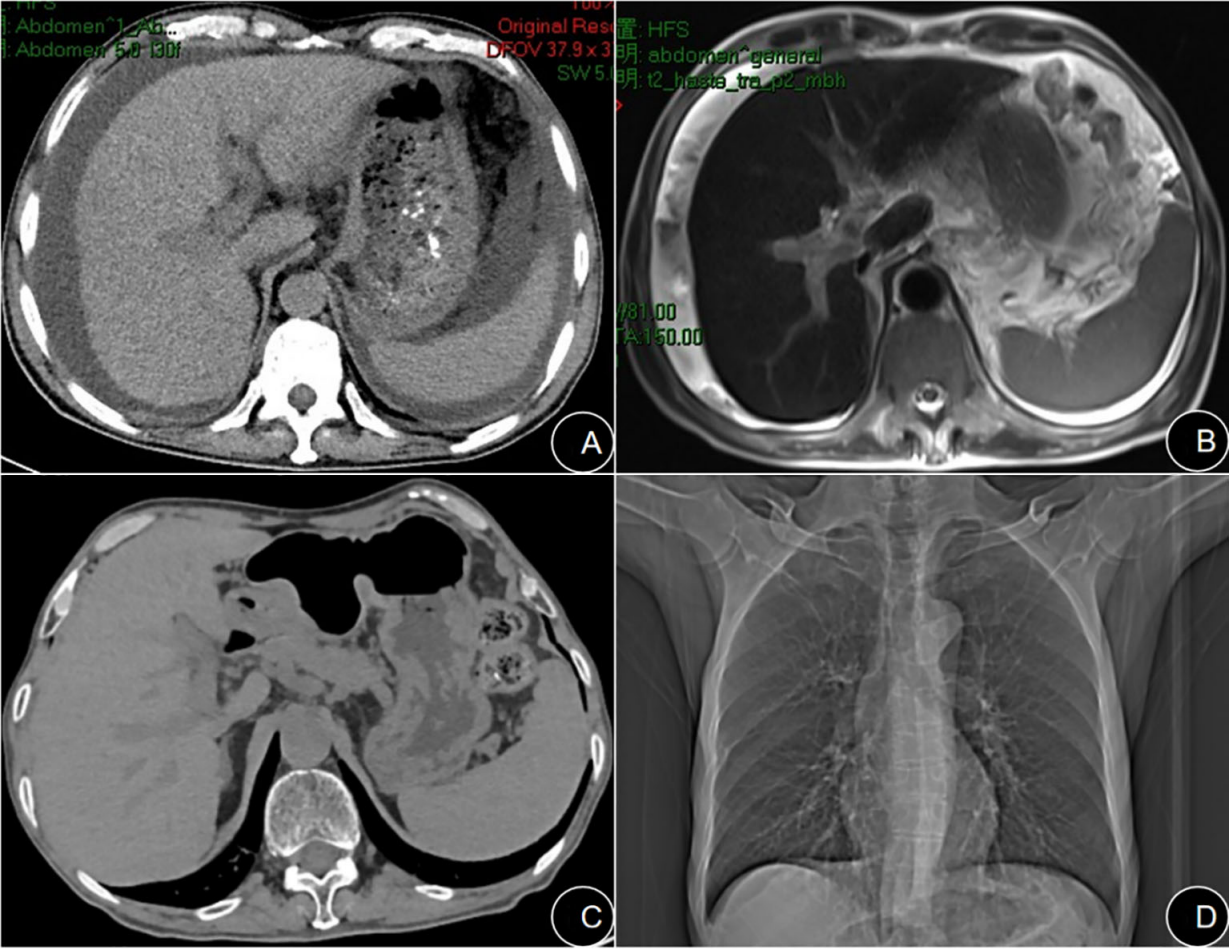
**(A)** Admission axial abdominal CT scan showing a widened portal vein diameter, massive ascites, and splenomegaly. **(B)** Admission T2-weighted abdominal MRI confirming ascites and heterogeneous liver parenchyma. **(C)** Follow-up axial abdominal CT scan after therapy demonstrates complete resolution of ascites and reduced splenic size. **(D)** Follow-up chest CT (bone window) reveals no osteolytic lesions.

**Figure 2:**
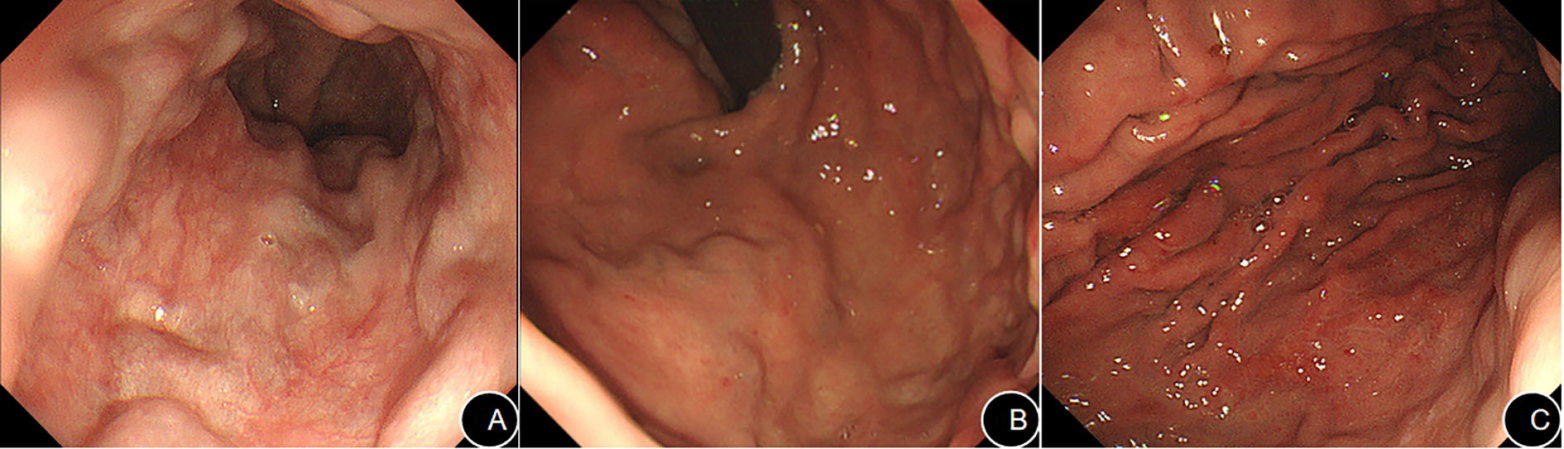
Endoscopic examination shows moderate-to-severe esophageal varices **(A)** and gastric fundal varices **(B)**, along with portal hypertensive gastropathy in the gastric body **(C)**.

On physical examination, the patient was conscious and alert, exhibiting hepatic facies with marked jaundice of the skin and sclerae. Stigmata of chronic liver disease, such as palmar erythema and spider angiomata, were absent. The abdomen was profoundly distended with epigastric tenderness upon palpation, but without rebound tenderness or muscular guarding. Shifting dullness was positive. The liver and spleen were not palpable due to the massive ascites. The remainder of the physical examination was unremarkable. Initial laboratory investigations revealed significant abnormalities in the complete blood count as well as in hepato-renal function (**Tables 1**, **2**). Serum Interleukin-6 (IL-6) was markedly elevated at 44.2 pg/mL (reference range: <7 pg/mL). Serological screening for Epstein-Barr virus (EBV), cytomegalovirus (CMV), rubella, and herpes simplex virus was negative. Analysis of the ascitic fluid showed a yellowish, slightly turbid appearance. The nucleated cell count was 160 cells/μL, with a predominance of mononuclear cells (90%). Biochemical analysis of the fluid revealed a low total protein level (19.9 g/L) and a low adenosine deaminase (ADA) level (6.63 U/L). Importantly, cytopathology and DNA ploidy analysis of the ascitic fluid identified no malignant cells. Imaging studies further delineated the extent of the disease. An abdominal computed tomography (CT) scan confirmed the presence of liver cirrhosis accompanied by massive ascites (**Figure 1A**). Magnetic resonance imaging (MRI) of the hepatobiliary system further characterized the cirrhotic morphology, demonstrating a shrunken liver with lobar disproportion and heterogeneous T1 signal intensity (**Figure 1B**). Esophagogastroduodenoscopy (EGD) verified the presence of moderate-to-severe esophagogastric varices and portal hypertensive gastropathy (**Figure 2**). Finally, transient elastography quantified liver stiffness at 20.6 kPa, a value consistent with grade F4 fibrosis, confirming established cirrhosis. The patient’s clinical course was complicated by refractory massive ascites. Despite repeated courses of diuretic therapy, albumin infusions, and therapeutic large-volume paracentesis (totaling 7640 mL), his abdominal distension persisted. To elucidate the etiology of his cirrhosis, common causes were systematically excluded; serological tests for antinuclear antibodies (ANA), the associated ANA profile, and the autoimmune hepatitis panel were all negative.

**Table 1:** Dynamics of hepato-renal function and serum albumin during different treatment phases.

Date	ALT (U/L) [9-50]	AST (U/L) [15-40]	ALP (U/L) [45-125]	GGT (U/L) [10-60]	TBil (μmol/L) [5-21]	DBil (μmol/L) [0-3.4]	ALB (g/L) [40-55]	Cr (μmol/L) [41-109]	Key events/treatment phase
2024-06-08	68	95	404	481	29.8	14.7	38.9	272	Baseline
2024-06-21	64	106	308	356	32.5	15.8	35.8	246	Conventional hepatoprotective therapy
2024-06-27	36	68	315	306	35.9	17.9	31.3	184	Pre-VCD regimen
2024-07-01	49	218	-	333	24.3	13.9	-	208	Acute liver injury on Day 3 of VCD
2024-07-07	76	65	388	401	23.0	11.6	29.6	161	During VCD therapy
2024-07-27	21	30	255	195	29.9	12.8	36.1	125	GI bleeding; VCD regimen terminated
2024-08-02	34	60	290	206	15.3	7.5	30.2	108	Daratumumab (Dara) therapy initiated
2024-08-14	30	34	415	274	22.0	9.1	34.3	98	Sustained remission
2024-09-25	14	20	271	255	21.7	7.5	33.0	97	Sustained remission
2024-10-23	20	24	243	337	14.3	5.4	35.7	81	Sustained remission
2025-04-18	62	34	203	110	9.9	3.7	39.9	90	Last follow-up

ALT, alanine aminotransferase; AST, aspartate aminotransferase; ALP, alkaline phosphatase; GGT, gamma-glutamyl transferase; TBil, total bilirubin; DBil, direct bilirubin; ALB, albumin; Cr, creatinine; GI, gastrointestinal.

**Table 2:** Dynamic changes in complete blood count parameters during different treatment phases.

Date	WBC(×10^9^/L) [3.5-9.5]	RBC(×10^12^/L) [4.3-5.8]	Hb(g/L) [130-175]	PLT(×10^9^/L) [100-300]	Key events/treatment phase
2024-06-08	4.17	3.5	112	172	Baseline
2024-06-12	5.46	3.24	107	169	Conventional hepatoprotective therapy
2024-06-27	5.73	3.69	125	130	Conventional hepatoprotective therapy
2024-07-01	10.14	3.62	126	85	During VCD therapy, thrombocytopenia occurred
2024-07-04	4.44	3.35	116	35	During VCD therapy
2024-07-10	3.66	2.96	99	44	During VCD therapy
2024-08-02	10.79	2.53	86	273	Dara therapy initiated
2024-08-07	7.22	3.15	107	204	During Dara therapy
2024-08-21	4.79	3.06	101.0	201	During Dara therapy
2024-09-25	7.30	3.15	98	308	During Dara therapy
2024-10-23	10.90	3.54	108	302	During Dara therapy
2024-11-06	7.60	3.38	100	299	During Dara therapy
2025-04-18	4.23	3.52	115	193	Last follow-up

WBC, white blood cells; RBC, red Blood Cells; Hb, hemoglobin; PLT, platelets.

**Figure 3:**
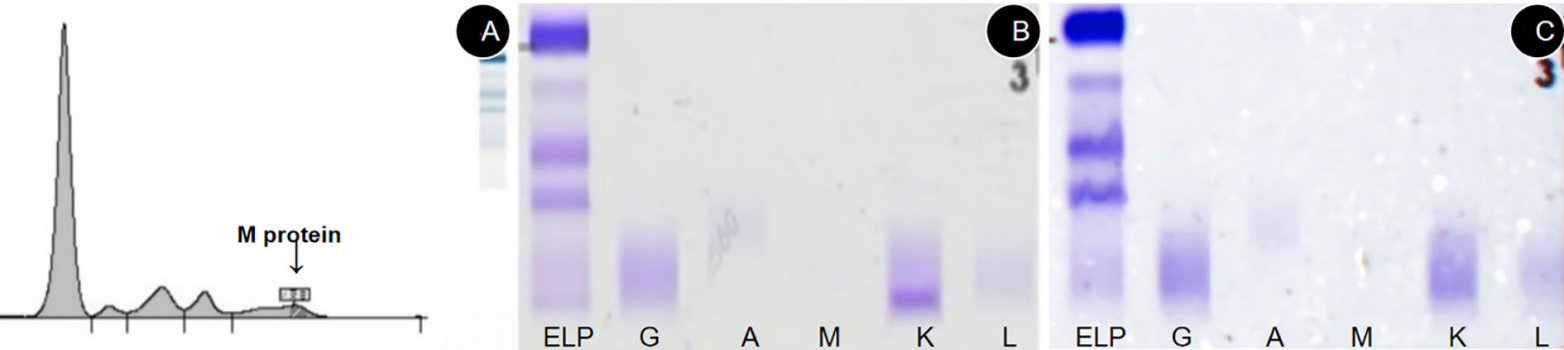
Electrophoresis Findings. On June 24, 2024, serum protein electrophoresis **(A)** showed a small M-protein; serum immunofixation electrophoresis **(B)** further confirmed the presence of an M-protein band in the electrophoretic lane, which formed a specific precipitation band with the anti-κ antibody, indicating a kappa-type monoclonal protein. On repeat serum immunofixation electrophoresis on April 18, 2025 **(C)**, no abnormal monoclonal bands were seen in any lane.

**Figure 4:**
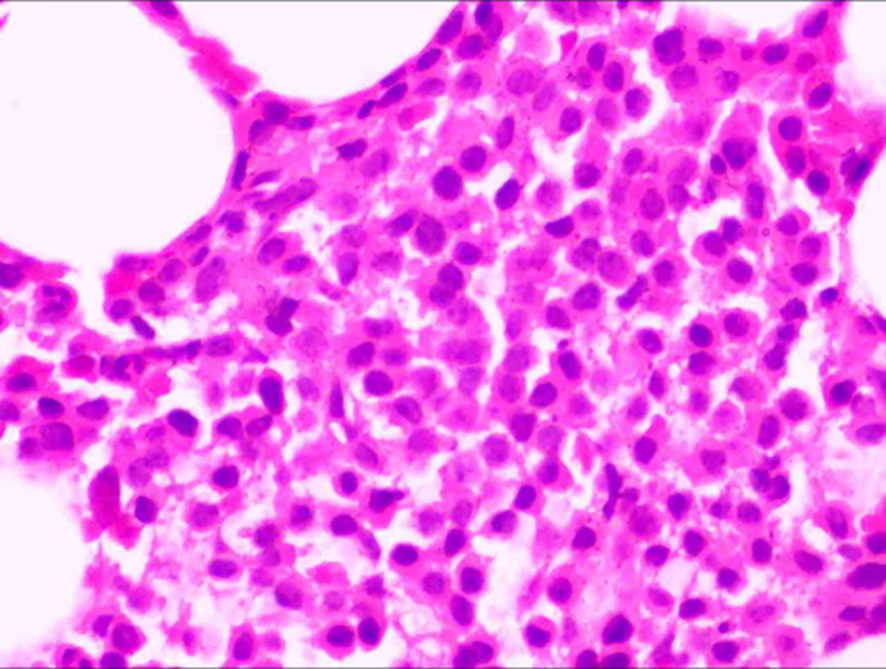
The bone marrow aspirate biopsy specimen demonstrated hyperplastic lymphohematopoietic tissue, occupying approximately 60% of the marrow space.

During this extensive workup, a constellation of anomalous immunological parameters was identified. Quantitative immunoglobulin analysis revealed profound hypogammaglobulinemia, with markedly decreased levels of IgG (5.4 g/L), IgA (0.44 g/L), and IgM (0.19 g/L). Correspondingly, serum protein electrophoresis (SPEP) showed elevated α1 (5.6%) and α2 (15.8%) globulin fractions with a decreased γ-globulin fraction (10.7%).These findings raised strong suspicion for an underlying plasma cell dyscrasia, prompting a consultation with the hematology service. Subsequent serum immunofixation electrophoresis (IFE) was positive for a monoclonal kappa (κ) light chain protein (**Figure 3**). The diagnosis was further solidified by the serum free light chain (sFLC) assay, which revealed an exceptionally high level of involved free κ light chains at 47,309.88 mg/L (reference range: 3.3-19.4 mg/L), while the uninvolved free λ light chain level was normal at 10.33 mg/L. This resulted in a severely abnormal sFLC ratio (κ/λ) of 4579.85 (reference range: 0.26-1.65). Additionally, serum beta-2 microglobulin (β2-M) was significantly elevated at 6,708 ng/mL (**Table 3**). Parallel urine studies confirmed renal involvement, showing nephrotic-range proteinuria (3.594 g/24h), a positive Bence-Jones protein test, and an elevated urine kappa light chain level of 379 mg/mL. To establish a definitive tissue diagnosis, a bone marrow aspiration and biopsy from the anterior superior iliac crest were performed. The aspirate smear showed an increased plasma cell population of 13.5%. The core biopsy, however, revealed a more extensive, near-total replacement of the marrow space with sheets of plasma cells, accounting for approximately 60% of total cellularity (**Figure 4**). Immunohistochemical staining demonstrated that these neoplastic plasma cells expressed a typical immunophenotype: positive for CD38 and CD138, but negative for CD19, CD20, and CD56, with monoclonal restriction for kappa light chains.

**Table 3:** Changes in serum free light chains (FLC) and beta-2 microglobulin (β2-MG) levels were analyzed before and after therapeutic intervention.

Parameter	Pre-treatment (2024-06-24)	Post-treatment (2024-11-21)	Reference Range
Serum Free κ Light Chains	47,309.88 mg/L	9.53 mg/L	3.30-19.40 mg/L
Serum Free λ Light Chains	10.33 mg/L	6.82 mg/L	5.71-26.30 mg/L
Serum κ/λ ratio	4,579.853	1.3974	0.26-1.65
Urinary Free κ Light Chains	3790mg/L	64.05 mg/L (2025-2-26)	1.17-86.46mg/L
Urinary Free λ Light Chains	**<**50mg/L	9.37 mg/L (2025-2-26)	0.27-15.21mg/L
Urinary κ/λ ratio	-	6.8356	1.83-14.26
β2-microglobulin	6,708 ng/mL	3441 ng/mL (2024-10-23)	1300–3000 ng/mL

Collectively, these findings supported the diagnosis of κ-LCMM. Prognostic evaluation via fluorescence *in situ* hybridization (FISH) analysis of bone marrow plasma cells revealed a deletion of the long arm of chromosome 13 (del(13q)); however, no high-risk cytogenetic abnormalities such as del(17p), IGH rearrangements (e.g. t(4;14), t(14;16)), or 1q21 amplification were detected. The patient was therefore classified as having Standard-Risk cytogenetics. A comprehensive radiographic skeletal survey revealed no lytic bone lesions.

The patient started first-line treatment with the VCD regimen (bortezomib 2 mg, cyclophosphamide 400 mg, dexamethasone 40 mg) on June 29, 2024. On the third day of treatment, the patient’s AST level acutely increased from 68 U/L to 218 U/L, suggesting drug-induced liver injury. During this period, his clinical symptoms, such as refractory ascites, did not improve. After one cycle of therapy, the patient was readmitted on July 27, 2024, due to hematemesis and melena, which was considered to be bleeding from portal hypertensive gastropathy. Given the patient’s intolerance to the VCD regimen and its lack of clinical efficacy, we decided to terminate this regimen. Considering the patient’s poor liver function reserve and inability to tolerate conventional combination chemotherapy, the treatment plan was adjusted to targeted monotherapy with daratumumab (16 mg/kg, 800 mg total, once weekly) after a multidisciplinary discussion (MDT). After three cycles of treatment, the severity of the patient’s ascites improved. A follow-up transient elastography examination showed a significant decrease in liver stiffness from 20.6 kPa to 15.1 kPa, with the fibrosis grade reversing from F4 to F3. Beta-2 microglobulin decreased from 6708 ng/mL to 3,441 ng/mL, Serum and urinary Free κ Light Chains level exhibited a significant decrease (**Table 3**), and liver and kidney function improved significantly (**Table 1**).

**Figure 5:**
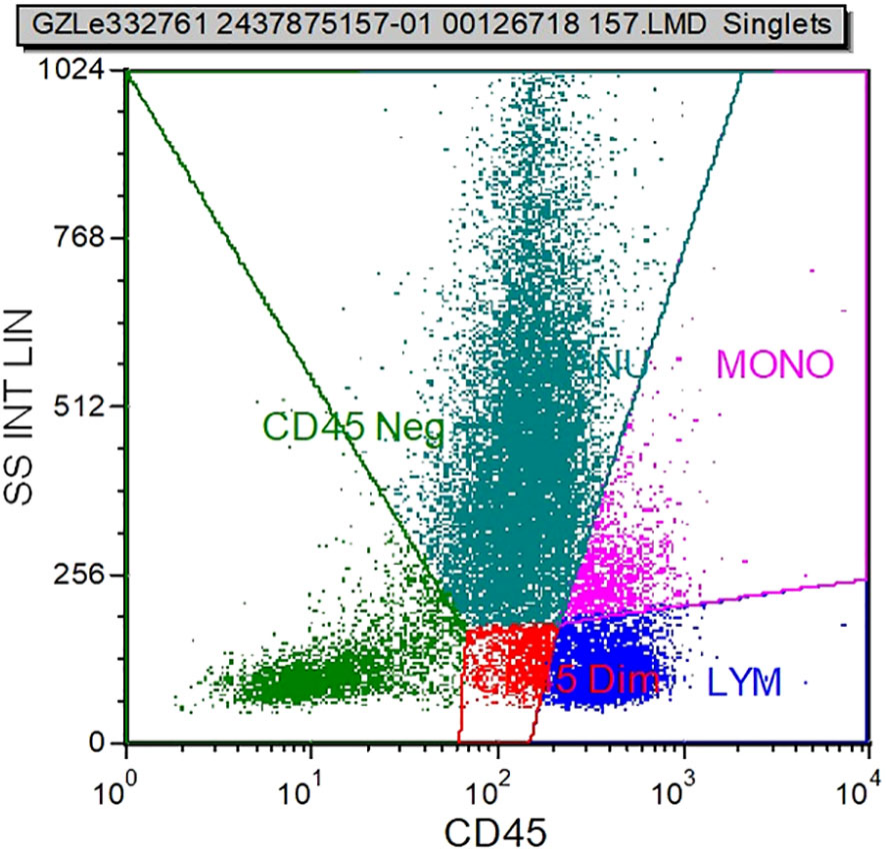
On April 9, 2025, bone marrow flow cytometry immunofluorescence analysis did not detect CD38+/CD138+ plasma cells, and both the plasma cell count and immunophenotype showed no abnormalities.

With continued treatment, by April 18, 2025 (the last follow-up), a follow-up abdominal CT (**Figure 1C**) indicated complete resolution of ascites, reduction in portal vein diameter compared to the previous scan, and decreased splenic volume; a concurrent chest CT (**Figure 1D**) revealed no lytic bone lesions. The patient underwent another bone marrow aspiration at this follow-up visit. Bone marrow cytology indicated that the marrow’s nucleated cells were actively proliferating, with a granulocyte-to-erythroid ratio of 4.36 and a granulocyte proportion of 69.8%, and no abnormalities in cell morphology were observed. Serum immunofixation electrophoresis showed that no abnormal monoclonal bands were found in any lane (**Figure 3C**). The results of bone marrow flow cytometry immunofluorescence analysis did not find phenotypically abnormal clonal plasma cells (CD38+/CD138+) (**Figure 5**).

## Discussion

κ-LCMM is a distinct subtype of multiple myeloma, characterized by the overproduction of abnormal immunoglobulin kappa chains. These abnormal light chains can deposit in tissues, causing organ damage. With the accelerating process of global population aging, the incidence of this disease shows a significant upward trend, with the elderly population over 65 years of age constituting the primary affected demographic (1). This case report describes a rare case of a patient with κ-LCMM presenting with refractory ascites and decompensated liver cirrhosis as the initial and primary manifestations. Upon initial admission, the patient lacked the typical “CRAB” symptoms (hypercalcemia, renal impairment, anemia, and bone destruction), which posed a significant challenge for early diagnosis and could easily have been misdiagnosed as common end-stage liver disease. A definitive diagnosis was only established after other causes of cirrhosis were systematically excluded and through subsequent serological and bone marrow pathology examinations. This case vividly highlights the significant heterogeneity of clinical manifestations in MM, particularly in the light-chain subtypes.

This case presents a profound diagnostic challenge by illustrating how multiple myeloma (MM) can masquerade as cryptogenic decompensated cirrhosis, an exceptionally rare and clinically deceptive initial presentation. The patient’s journey underscores the critical importance of maintaining a broad differential diagnosis when confronted with unexplained organ failure.

The pathogenesis of the patient’s refractory ascites and hepatic dysfunction is best understood through a ‘multiple-hit’ model. First, the immense serum free kappa light chain burden (47,309.88 mg/L) posed a direct risk for hepatic toxicity and injury, potentially establishing a baseline of portal hypertension. Second, concurrent renal light chain excretion led to nephrotic-range proteinuria (3.594 g/24h), inducing profound hypoalbuminemia. Consequently, the refractory ascites emerged from the synergy of increased portal pressure and critically reduced plasma oncotic pressure. The definitive validation of this model lies in the response to daratumumab. By eliminating the monoclonal light chain source, therapy simultaneously ameliorated both processes, evidenced by ascites resolution and a precipitous drop in serum and urine kappa light chains. While liver stiffness regression is consistent with this trend, its interpretation is tempered by the known confounding effect of ascites. The assessment of portal hypertension reversal would have been strengthened by follow-up Doppler ultrasound or endoscopy, which were not performed. Nevertheless, the unequivocal resolution of ascites on CT remains the cornerstone objective evidence, highlighting that effective treatment hinges on targeting the root plasma cell clone.

From a pathophysiological perspective, systemic mechanisms inherent to MM biology may have contributed to a pro-fibrotic hepatic milieu. The PI3K/Akt/mTOR signaling pathway, which is upregulated in MM and central to tumor proliferation (2, 3), is also known to activate hepatic stellate cells (HSCs) and promote hepatic fibrogenesis (4). It is plausible that paracrine signaling from plasma cells or systemic inflammation could have engaged this pathway in the liver. Furthermore, the elevated serum IL-6 level (44.2 pg/mL) observed in our patient—a cytokine whose level correlates with MM disease stage (5, 6)—may have provided a persistent pro-fibrotic stimulus. IL-6 is a potent activator of HSCs, driving their transformation into collagen-producing myofibroblasts via pathways like JAK/STAT and MAPK, thereby accelerating fibrosis (7). Thus, while not proven, these MM-associated pathways represent plausible contributors to the initiation or progression of liver fibrosis in the context of an overwhelming light chain burden.

A review of the literature indicates that hepatic involvement in MM presents in various forms, including light chain deposition disease, plasmacytoma, amyloidosis, or diffuse hepatic infiltration (8). The deposition of a large volume of κ-light chains in the liver, for example, causes architectural distortion and cirrhosis (9, 10). A separate but related mechanism involves the deposition of amyloid or light chains within the hepatic sinusoidal spaces, which can cause non-cirrhotic portal hypertension by obstructing blood flow, as reported by Su et al. (11). Both pathways culminate in the common clinical endpoint of portal hypertension and ascites observed in our patient. The consistently poor survival associated with hepatic involvement in MM (12) underscores the aggressiveness of these processes. In the present case, a liver biopsy was difficult to perform due to the massive ascites, and thus, pathological evidence from the liver tissue could not be obtained. In future clinical practice, for similar patients for whom conditions permit, relatively safer biopsy methods, such as a transjugular liver biopsy, should be considered to obtain a pathological diagnosis.

Light chain deposition disease (LCDD) is a rare hematologic disorder, primarily characterized by the deposition of non-amyloid monoclonal light chains within multiple organs. Among the affected organs, the kidneys are the principal site of deposition, with other sites including the hepatic sinusoids, choroid plexus, or myocardium (13). When the liver is involved, the primary manifestations include mild to moderate elevation of transaminases, portal hypertension, or fulminant hepatic failure (13, 14). The abnormal transaminases and manifestations of portal hypertension in the present case are consistent with those reported in the literature. Additionally, the heart is another commonly affected site in LCDD, with potential manifestations including arrhythmias, restrictive cardiomyopathy, or even an atrial mass (15). Given this, when conducting an initial patient assessment, one should ensure it encompasses a detailed medical history, a comprehensive physical examination, and a thorough laboratory evaluation to gain as complete an understanding of the patient’s condition as possible, thereby providing a reliable basis for an accurate diagnosis and appropriate treatment.

Despite conventional treatments for decompensated cirrhosis, such as hepatoprotective therapy, sodium-restricted diuretics, ascites drainage, and human serum albumin infusions, the patient’s hepato-renal function and ascites showed no significant improvement. It was not until after the initiation of MM-specific therapy that the aforementioned symptoms improved significantly. Elderly patients with MM often present with multiple comorbidities or organ dysfunction; therefore, selecting a therapeutic regimen with low toxicity that is also effective is critically important. As a fully human anti-CD38 IgGκ monoclonal antibody, daratumumab can specifically bind to and induce cytolytic cell death (16). Compared with the traditional VCD regimen, the daratumumab regimen demonstrated a dual advantage in this case: it directly targets CD38+ plasma cells, rapidly eliminating the source of pathogenic light chains; and its characteristic of not undergoing hepatic metabolism makes it particularly suitable for patients with impaired liver function. The profound efficacy of daratumumab in achieving deep hematologic responses and reversing organ damage is strongly supported by evidence from large clinical trials. In the ANDROMEDA trial, adding daratumumab to bortezomib, cyclophosphamide, and dexamethasone significantly improved hematologic complete response rates and prolonged survival free from major organ deterioration or hematologic progression in patients with newly diagnosed AL amyloidosis, with a manageable safety profile (17). A study by Kastritis et al. (18) has confirmed the high efficacy of daratumumab in the treatment of LCDD. The significant improvement in both clinical symptoms and organ function in our patient after receiving daratumumab therapy provides strong clinical evidence for the superiority of this therapeutic regimen.

## Conclusion

This case highlights that for patients with unexplained cirrhosis accompanied by decreased immunoglobulin levels, clinicians should include plasma cell dyscrasias in the differential diagnosis. Early screening with serum free light chain assays and bone marrow examination is key to avoiding misdiagnosis and improving prognosis. Furthermore, this paper has also elaborated on the potential mechanisms by which MM may lead to cirrhosis and has proposed corresponding therapeutic strategies. Currently, our understanding of the specific mechanisms by which MM causes cirrhosis remains incomplete. More in-depth basic research is needed to reveal the exact pathophysiological processes in order to provide more precise therapeutic strategies for this patient population.

## Data Availability

The original contributions presented in the study are included in the article/supplementary material. Further inquiries can be directed to the corresponding author/s.
